# Semantic-enhanced heterogeneous graph learning for identifying ncRNAs associated with drug resistance

**DOI:** 10.1093/bioinformatics/btag029

**Published:** 2026-01-14

**Authors:** Hang Wei, Yuran Xie, Wenxiang Zhang, Linyang Li, Shuai Wu, Lin Gao

**Affiliations:** School of Computer Science and Technology, Xidian University, Xi’an, Shaanxi 710126, China; School of Computer Science and Technology, Xidian University, Xi’an, Shaanxi 710126, China; School of Biomedical Engineering, Shenzhen University Medical School, Shenzhen 518060, China; Central Laboratory, Shenzhen University General Hospital, Shenzhen University, Shenzhen 518060, China; School of Computer Science and Technology, Xidian University, Xi’an, Shaanxi 710126, China; School of Computer Science and Technology, Xidian University, Xi’an, Shaanxi 710126, China; School of Computer Science and Technology, Xidian University, Xi’an, Shaanxi 710126, China

## Abstract

**Motivation:**

Identifying non-coding RNAs (ncRNAs) associated with drug resistance is critical for elucidating molecular mechanisms underlying drug response, facilitating drug screening, and discovering novel therapeutic targets. While several graph neural network-based methods have been proposed to infer ncRNA-drug resistance associations, they remain fundamentally constrained by semantic distortion induced by a sparse bipartite network and neglect of relational semantics among molecular entities, ultimately compromising both predictive reliability and biological interpretability.

**Results:**

In this study, we propose iNcRD-HG, a novel framework for identifying ncRNA-drug resistance associations. The framework addresses three critical aspects: constructing a context-enriched heterogeneous network that integrates six distinct molecular interaction types with bio-entity-specific attributes, developing a semantic-enhanced graph learning architecture that implements relation-type-aware message passing to capture complex contextual dependencies, and introducing an interpretability mechanism to reveal potential synergistic pathways underlying drug response. Experimental results demonstrate that iNcRD-HG achieves superior predictive performance across diverse benchmark datasets while deriving association features with strong discriminative capability. By identifying molecular synergistic contexts, iNcRD-HG provides mechanistically interpretable insights into ncRNA-mediated drug resistance.

**Availability and implementation:**

Datasets and source codes are available at https://github.com/Biohang/iNcRD-HG.

## 1. Introduction

Chemotherapeutic drugs serve as a cornerstone in the treatment of diseases, especially for malignant tumors. However, their therapeutic efficacy is frequently compromised by challenges such as tumor recurrence or progression post-treatment in most patients. A pivotal factor underlying therapeutic failure is the emergence of drug resistance, a multifactorial process orchestrated through diverse mechanisms like drug efflux pumps, target alterations, DNA repair enhancement, tumor microenvironment remodelling, and bypass alternative signalling pathway activation (Khan *et al.* 2024, Lin 2024, Patel *et al.* 2024, Yan *et al.* 2024, Qi *et al.* 2025, Qiao *et al.* 2025). This resistance phenotype stems from the crosstalk between molecular pathways, which collectively enable tumor cell survival under pharmacological pressure (Musyuni *et al.* 2022).

Non-coding RNAs (ncRNAs), as critical regulators of cellular processes, have been well-documented to participate in diverse physiological and pathological activities (Tang *et al.* 2018, Nemeth *et al.* 2024, Qiao *et al.* 2024). Recent studies have increasingly highlighted their fundamental involvement in mediating drug resistance (Chen *et al.* 2024, Wang *et al.* 2024). For examples, miR-23a-3p upregulation in an ETS1-dependent manner promotes sorafenib resistance in HCC, whereas its CRISPR-Cas9 knockout restores drug sensitivity (Lu *et al.* 2022). In multiple myeloma, H19 drives bortezomib resistance via the miR-29b-3p/MCL-1 axis (Pan *et al.* 2019). Identifying ncRNA-mediated drug resistance mechanism is critical for facilitating drug screening, developing tailored strategies against resistance, and discovering novel druggable targets within ncRNA-mediated regulatory networks.

The growing accumulation of biological data enables computational methods that exploit prior associations to identify ncRNA associated with drug resistance, offering guidance for experimental validation (Mahapatra *et al.* 2024, Chen *et al.* 2025, Lai *et al.* 2025). These methods fall into two main categories: pan-ncRNA predictors and models tailored to specific ncRNA types. Most pan-ncRNA predictors regard diverse ncRNAs as homogeneous nodes and leverage prior ncRNA–drug resistance associations within graph learning frameworks. Their main challenge is the highly sparse supervision signals of the prior bipartite network. GSLRDA addressed this by integrating dropout and random walk–based contrastive networks with lightweight GCN and self-supervised learning to enhance node representations (Zheng *et al.* 2022, Ren *et al.* 2025). NDSGCL further integrated local structural and global semantic neighbours for data augmentation (Hu *et al.* 2024), while AGCLNDA introduced adaptive graph contrastive learning by constructing enhanced and denoised bipartite graphs to improve prediction performance (Fan *et al.* 2025). Computational methods focusing on specific ncRNA types, primarily miRNAs and lncRNAs, have been developed, with research on miRNA–drug resistance garnering more attention. GCMDR integrated miRNA expression and functional similarity to represent miRNA attributes and used drug substructure fingerprints to characterize drugs, leveraging GCN to uncover potential associations from a heterogeneous miRNA–drug association network (Huang *et al.* 2020). SDNEMDA combined BiGRU and SDNE to integrate node attribute and topological features (Wang *et al.* 2024, Lei and Lei 2025), while DLST-MDA employed multi-scale CNN sequence encoder and GCN structure encoder to capture high-level miRNA and drug representations (Sheng *et al.* 2025). In contrast, research on lncRNA-drug resistance remains limited. DeepLDA employed the GIP strategy to construct lncRNA and drug similarity networks and utilized GCN and GAT to extract high-level representations, achieving promising predictive performance (Wei *et al.* 2021, Gao and Shang 2023, Yan *et al.* 2023, Ai *et al.* 2024).

Existing graph learning–based computational methods have advanced our understanding of the roles of ncRNAs in drug resistance mechanisms. Nevertheless, several challenges remain. (i) Although pan-ncRNA predictors design different contrastive learning frameworks to alleviate sparsity and noise in the original bipartite network, they still rely heavily on limited prior signals. Consequently, the biological semantic information embedded in the constructed contrastive networks is restricted and prone to semantic distortion. (ii) Whether pan-ncRNA predictors or models tailored to specific ncRNA types, most approaches focus exclusively on bipartite associations between individual ncRNAs and drugs. In reality, ncRNAs exert their functions via intricate molecular interactions that impact drug efficacy. Current methods overlook these synergistic ncRNA interactions, yielding networks devoid of molecular interaction context and unable to capture drug response-related molecular pathways. Overall, integrating biological contextual semantics to improve predictive reliability and interpretability remains a critical challenge (Liu *et al.* 2024).

To investigate the multifaceted roles of ncRNAs in drug resistance mechanisms, we propose a novel pan-ncRNA predictor, iNcRD-HG, for predicting ncRNA–drug resistance associations. iNcRD-HG enhances the biological semantics of prior sparse network by incorporating multi-source biological information from both node attributes and topological structures. Specifically, it integrates cooperative ncRNA interactions, intra-type molecular associations, specific expression profiles of ncRNAs across diverse cancer cell lines, and drug Simplified Molecular-Input Line-Entry System (SMILES) to construct a heterogeneous biological network. On this basis, we design a semantics-enhanced heterogeneous graph learning architecture that employs relation-type–specific modules with dynamic message passing to capture semantic dependencies between nodes, while incorporating an interpretability mechanism to infer biologically meaningful paths underlying predictions. Experimental results indicate that the constructed heterogeneous network effectively encodes rich biological semantics, enabling iNcRD-HG to achieve more reliable predictions of ncRNA–drug resistance associations.

## 2. Materials and methods

### 2.1 Datasets

The experimentally validated ncRNA–drug resistance associations were retrieved from ncRNADrug (Cao *et al.* 2024). To better encode the attributes of ncRNAs and drugs, ncRNA expression profiles were extracted from CCLE (Barretina *et al.* 2012, Ghandi *et al.* 2019), and drug SMILES strings were obtained from PubChem (Kim *et al.* 2025). Duplicate entries in ncRNADrug, along with ncRNAs and drugs lacking expression profiles or SMILES strings, were excluded. Following preprocessing, we obtained 1322 lncRNAs, 605 miRNAs, 216 drugs, 2109 lncRNA–drug resistance associations, and 5394 miRNA–drug resistance associations. The resulting ncRNA–drug resistance association datasets utilized for model training and evaluation can be represented as follows:


(1) $$\{\begin{array}{l} \;S_{\mathrm{LD}}=S_{\mathrm{LD}}^{+}\cup S_{\mathrm{LD}}^{-}\\ {\;S}_{\mathrm{MD}}=\;S_{\mathrm{MD}}^{+}\cup S_{\mathrm{MD}}^{-} \end{array}$$

where $S_{\mathrm{LD}}^{+}$ and $S_{\mathrm{MD}}^{+}$ denote the known lncRNA-drug and miRNA-drug resistance associations, respectively. $S_{\mathrm{LD}}^{-}$ and $S_{\mathrm{MD}}^{-}$ represent negative pairs randomly generated from distinct extensive sets of unknown pairs, respectively, with their quantities being twice those of the corresponding positive associations. The dataset $S_{type}^{+}$ and $S_{type}^{-}\;(type\in \{\mathrm{LD},\;\mathrm{MD}\})$ can be further partitioned into training, validation, and testing sets in 0.6:0.2:0.2 ratio, as described below:


(2) $$\left\{ \begin{array}{c} \begin{array}{l} \begin{array}{l} S_{type}^{+}=S_{type}^{\mathrm{train}+}\cup S_{type}^{\mathrm{val}+}\cup S_{type}^{\mathrm{test}+}\\ S_{type}^{-}=S_{type}^{\mathrm{train}-}\cup S_{type}^{\mathrm{val}-}\cup S_{type}^{\mathrm{test}-} \end{array}\\ S_{type}^{\mathrm{train}}=S_{type}^{\mathrm{train}+}\cup S_{type}^{\mathrm{train}-} \end{array}\\ \begin{array}{l} S_{type}^{\mathrm{val}}=S_{type}^{\mathrm{val}+}\cup S_{type}^{\mathrm{val}-}\\ S_{type}^{\mathrm{test}}=S_{type}^{\mathrm{test}+}\cup S_{type}^{\mathrm{test}-} \end{array} \end{array}\right.$$

where training set $S_{type}^{\mathrm{train}}$ is used to train model, validation set $S_{type}^{\mathrm{val}}$ is employed for hyperparameter tuning, and testing set $S_{type}^{\mathrm{test}}$ is reserved for final performance evaluation. No overlapping ncRNA–drug resistance pairs exist among the three subsets. Detailed statistical information for each sub-dataset is provided in Supplementary Table S1, available as supplementary data at *Bioinformatics* online. The datasets can be accessed and downloaded from https://zenodo.org/records/16919628.

### 2.2 The iNcRD-HG model architecture

**Figure 1:**
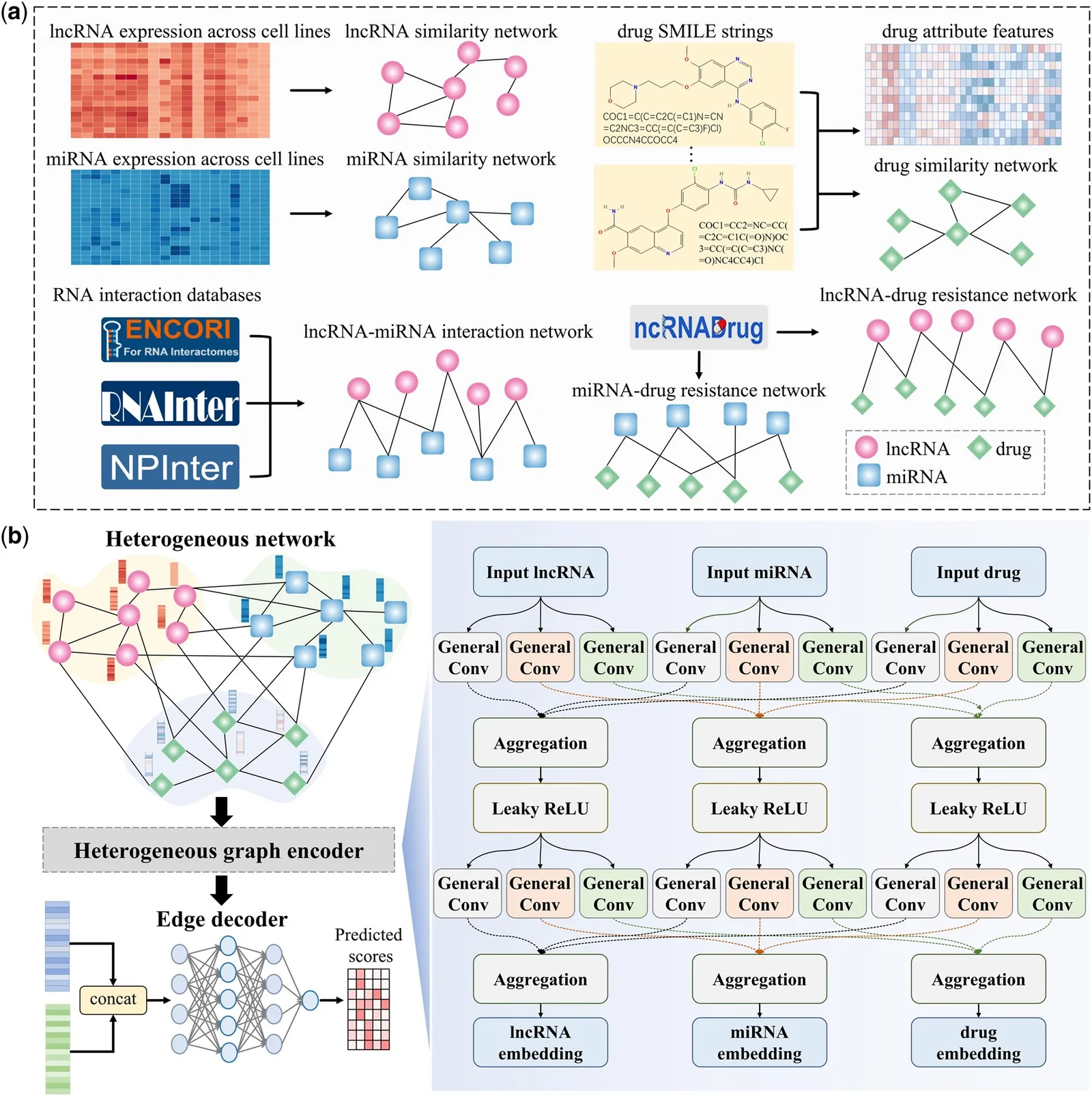
The architecture of iNcRD-HG. (a) Heterogeneous network construction. The heterogeneous network is constructed based on ncRNA expression profiles, drug SMILES strings, validated ncRNA-drug resistance associations, and lncRNA-miRNA interactions. (b) Context-enriched heterogeneous graph learning. Node representations of ncRNAs and drugs are learned through the heterogeneous graph encoder. The edge decoder generates pairwise representations by concatenating ncRNA and drug features and predicts association scores using a multi-layer perceptron.

iNcRD-HG is a graph-learning based framework to predict pan-ncRNA-drug resistance associations. As illustrated in Fig. 1, it comprises two main modules: heterogeneous network construction and context-enriched heterogeneous graph learning. The details of each module are described in the following sections.

### 2.3 Heterogeneous network construction

To enrich the biological semantics of original ncRNA-drug resistance bipartite network, we performed attribute feature encoding for ncRNAs and drugs and constructed multi-relational subnetwork, thereby forming the heterogeneous association network shown in Fig. 1a.

To characterize ncRNAs within pathological contexts, we extracted expression profiles of miRNAs and lncRNAs across 938 cancer cell lines from CCLE (Barretina *et al.* 2012, Ghandi *et al.* 2019) and used them as initial node features for ncRNAs. For drugs, we encoded molecular structures using SMILES strings and derived their representations with ChemBERTa (Chithrananda *et al.* 2020), a transformer-based model pretrained on 77 million SMILES strings from PubChem (Kim *et al.* 2025) through masked language modeling and multi-task regression, enabling the capture of rich and generalizable chemical semantics.

Considering the relationships between different molecules, we constructed six multi-relational subnetworks, including lncRNA similarity network, miRNA similarity network, drug similarity network, lncRNA-miRNA interaction network, miRNA-drug resistance association network, and lncRNA-drug resistance association network. The ncRNA similarity networks were derived using Kendall’s tau coefficient based on the 938-dimensional expression profiles of miRNAs and lncRNAs. Drug similarity was calculated using the Tanimoto coefficient applied to drug SMILES strings. To ensure the high quality of the similarity networks, only edges with similarity scores greater than 0.5 were retained across all similarity networks. The lncRNA-miRNA interaction network was constructed by integrating experimentally validated interactions from RNAInter (Kang *et al.* 2022), NPInter (Zheng *et al.* 2023), and ENCORI (Li *et al.* 2014). The two ncRNA-drug resistance association networks were built using the curated prior associations in $S_{\mathrm{LD}}^{+}$ and $S_{\mathrm{MD}}^{+}$, retrieved from the ncRNADrug (Cao *et al.* 2024). Table 1 presents the statistical information of each subnetwork comprising the overall heterogeneous network.

**Table 1:** The statistic information of each subnetwork.

Subnetwork type	#Node 1	#Node 2	#Edge
lncRNA similarity	1322 lncRNAs	1322 lncRNAs	8772
miRNA similarity	605 miRNAs	605 miRNAs	16232
drug similarity	216 drugs	216 drugs	852
lncRNA-miRNA	1322 lncRNAs	605 miRNAs	1841
miRNA-drug resistance	605 miRNAs	200 drugs	5394
lncRNA-drug resistance	1322 lncRNAs	71 drugs	2109

### 2.4 Context-enriched heterogeneous graph learning

#### 2.4.1 Heterogeneous graph encoder

As heterogeneous graph learning excels at extracting rich semantics from complex biological networks (Liu *et al.* 2019, Yu *et al.* 2024, Zhang *et al.* 2024, Su *et al.* 2025), we developed a heterogeneous graph encoder based on relation-type-aware message passing modules for capturing multi-relational structure semantic of the constructed heterogeneous network. Different from standard Graph Convolutional Networks (GCN) layer (Kipf 2017, Li *et al.* 2021), General Convolution (GeneralConv) separately learns self-node and neighbour-specific transformation weights (You *et al.* 2020), enabling more flexible and expressive feature integration in noisy biological networks. Therefore, general convolution was used as the base operator for node representation learning. Formally, for each graph, the update rule is:


(3) $$h_{v}^{\left(l+1\right)}=\sigma (W^{\mathrm{self}}h_{v}^{\left(l\right)}+\sum_{u\in N(v)}W^{\mathrm{neigh}}h_{u}^{\left(l\right)})$$

where $h_{v}^{\left(l\right)}$ denotes the representation of node v at layer l, N(v) is its neighbourhood, $W^{\mathrm{self}}$ and $W^{\mathrm{neigh}}$ are learnable weights for self and neighbour contributions, respectively, and σ(·) denotes the LeakyReLU activation function.

There are six undirected edge types R={ll, mm, dd, lm, ld, md} in the constructed heterogenous network, where ll, mm, dd, lm, ld, md represent lncRNA similarity, miRNA similarity, drug similarity, lncRNA-miRNA interaction, lncRNA-drug association, miRNA-drug association, respectively. To exploit the semantic diversity, we extended general convolution into relation-type-aware modules. For each edge type r∈R, a relation-type-aware message function is defined as:


(4) $$f_{r}(v)=W_{r}^{\mathrm{self}}h_{v}^{\left(l\right)}+\;\sum_{u\in N_{r}(v)}W_{r}^{\mathrm{neigh}}h_{u}^{\left(l\right)})$$

where $N_{r}(v)$ denotes neighbours of v under relation r, and $W_{r}^{\mathrm{self}}$ and $W_{r}^{\mathrm{neigh}}$ are relation-type-aware learnable parameters. Thus, for each node type, the updated embeddings are computed as:


(5) $$\begin{array}{c} h_{\mathrm{lncRNA}}=\sigma (⊕(f_{\mathrm{ll}},f_{\mathrm{lm}},f_{\mathrm{ld}}))\\ h_{\mathrm{miRNA}}=\sigma (⊕(f_{\mathrm{mm}},f_{\mathrm{ml}},f_{\mathrm{md}}))\\ h_{\mathrm{drug}}=\sigma (⊕(f_{\mathrm{dd}},f_{\mathrm{dl}},f_{\mathrm{dm}})) \end{array}$$

where $f_{xy}$ denotes the message update function along relation <*x*-*y*>, ⊕ denotes the aggregation operator, which can be instantiated using pooling functions such as max, mean, sum, or min. By employing relation-type-aware general convolution modules, heterogeneous graph encoder dynamically models both intra-type similarities and cross-type dependencies. This semantic augmentation enables deeper integration of molecular contexts and enhances the expressiveness of ncRNA and drug embeddings.

#### 2.4.2 NcRNA-drug resistance association prediction

The edge decoder was designed to predict the ncRNA-drug resistance association scores. Through the heterogeneous graph encoder, we obtained high-level embeddings for lncRNAs, miRNAs, and drugs. Taking miRNA–drug resistance association prediction as an example, for a target miRNA–drug pair, a concatenation pooling layer was applied to capture the joint association features:


(6) $$h_{m,d}=\mathrm{concat}(h_{\mathrm{miRNA}},h_{\mathrm{drug}})$$

Subsequently, a multilayer perceptron (MLP) with three fully connected layers was employed to predict the association score:


(7) $$P={W_{3}\cdot \mathrm{Leaky}\_\mathrm{ReLU}(W}_{2}\cdot \mathrm{Leaky}\_\mathrm{ReLU}(W_{1}H_{m,d}+b_{1})+b_{2})+b_{3}$$

where $W_{1}$, $W_{2}$ and $W_{3}$ are three learnable parameter matrices, and $H_{m,d}$ is the miRNA-drug pair feature matrix. Concatenation pooling allows direct integration of miRNA and drug embeddings, preserving their distinct feature spaces, while the MLP captures complex nonlinear interactions between them, enabling more accurate modeling of association patterns. The binary cross-entropy is defined as the loss function for training iNcRD-HG:


(8) $$L=-\frac{1}{N}\sum_{i=1}^{N}\left[y_{i}\cdot \log s(p_{i})+(1-y_{i})\cdot \log\left(1-s(p_{i})\right)\right]$$

where *N* is the number of training pairs, s(·) is the sigmoid activation. $p_{i}$ and $y_{i}\in \{0,1\}$ denotes the predicted association score and ground truth label, respectively.

### 2.5 Performance evaluation

Two metrics including Area Under the Receiver Operating Characteristic Curve (AUC) and Area Under the Precision–Recall Curve (AUPR) are used to comprehensively evaluate the performance of different predictors (Guo *et al.* 2024, Huang *et al.* 2024, Zhu *et al.* 2024, Dao *et al.* 2025). In addition, because top-ranked predictions are typically more reliable and practically relevant, we also incorporate three top-k ranking metrics: Precision@k, NDCG@k, and Recall@k, which respectively evaluate the precision, normalized discounted cumulative gain (NDCG), and recall within the top-k predictions, providing a more fine-grained evaluation of predictive performance in practical settings. Detailed descriptions of these evaluation metrics are provided in the Supplementary Method, available as supplementary data at *Bioinformatics* online.

## 3. Results and discussion

### 3.1 Parameter sensitivity analysis for robustness evaluation

**Figure 2:**
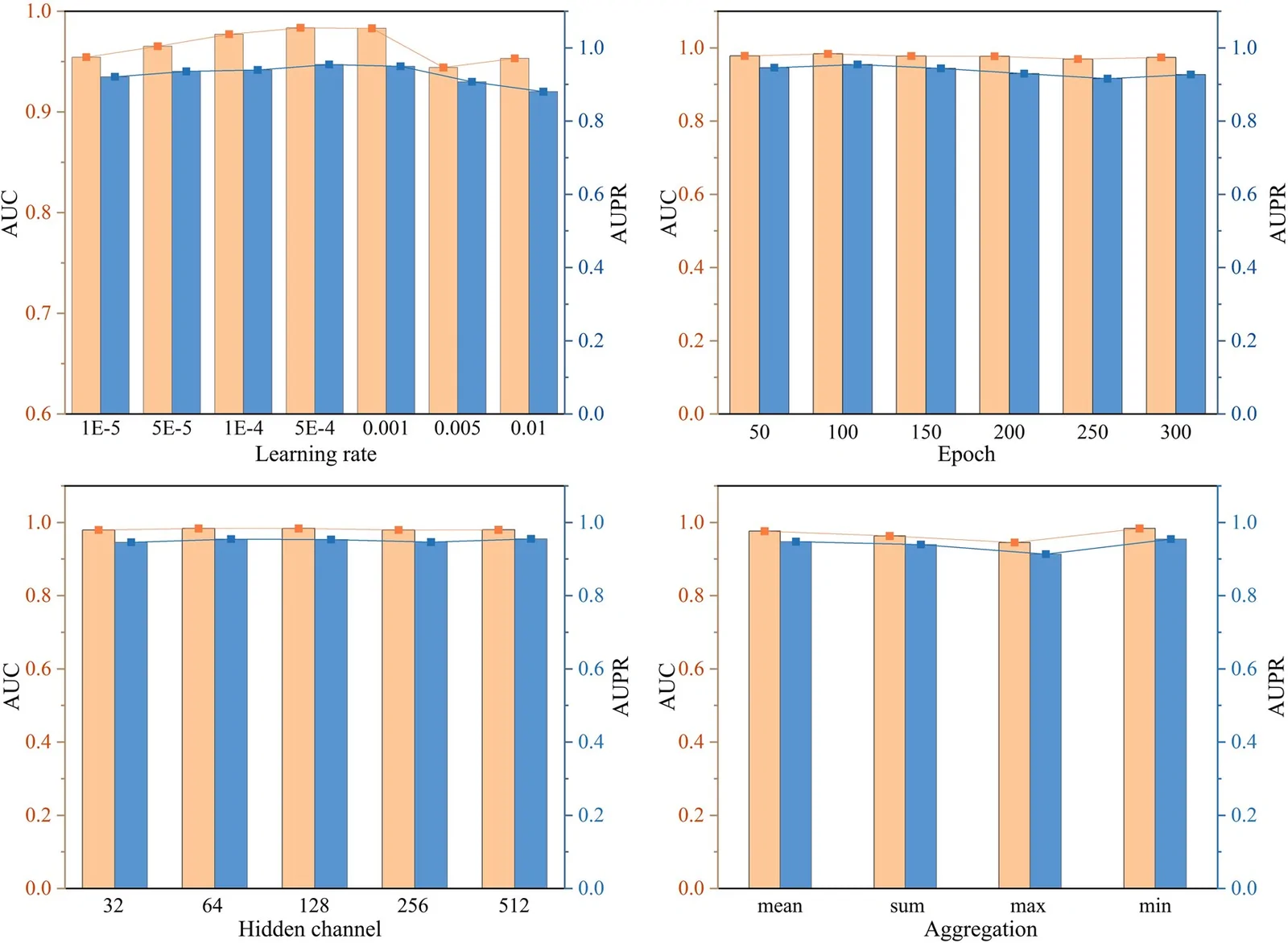
Parameter analysis of iNcRD-HG. AUC and AUPR obtained with different learning rates, training epochs, hidden dimensions, and aggregation strategies on $S_{\mathrm{LD}}^{\mathrm{val}}$.

To evaluate the robustness of iNcRD-HG, parameter sensitivity analysis was performed focusing on four critical hyperparameters: the learning rate, the number of training epochs, the hidden channel dimension in heterogeneous graph learning, and the aggregation strategy for message passing across different edge types. This analysis was conducted using the validation dataset $S_{\mathrm{LD}}^{\mathrm{val}}$, with the lncRNA–drug resistance association prediction task as an example. The results are presented in Fig. 2, revealing the following observations: (1) The learning rate is found to exert relatively pronounced effect on training iNcRD-HG. A low learning rate results in slow convergence and susceptibility to local optima, whereas an excessively high rate causes unstable training and impedes convergence to a satisfactory global optimum. Thus, selecting an appropriate learning rate is essential for effective and stable training; (2) Regarding the number of epochs and hidden channel dimension, iNcRD-HG performs relatively stable within suitable ranges, underscoring its resilience to such variations; (3) For the aggregation strategies employed in message passing across multiple edge types, we observe that the max pooling strategy consistently yields inferior performance. This can likely be attributed to its tendency to diminish the contribution of relation-type-aware semantic information. By contrast, the mean, sum, and min aggregation strategies produce comparable results, indicating that their influence on the overall predictive performance is largely interchangeable. Collectively, these findings indicate that iNcRD-HG exhibits strong robustness to variations in its key hyperparameters. Based on a grid search, we subsequently determined the optimal configuration for the lncRNA–drug resistance association prediction task as follows: {learning rate = 0.0005, epochs = 100, hidden channel dimension = 64, aggregation strategy=’min’}. In addition, the same parameter search space was applied to miRNA–drug resistance association prediction task, with the corresponding results are presented in Supplementary Fig. S1, available as supplementary data at *Bioinformatics* online.

### 3.2 Semantic enrichment boosts predictive capability

To evaluate the effectiveness of the semantic enrichment strategy, which combines attribute feature encoding for ncRNAs and drugs with the construction of multi-relational subnetworks to enhance the initially sparse prior association network, we conducted ablation experiments to compare iNcRD-HG with three baseline variants: w/o_LncMi, w/o_attribute and HG_SeqSim.

The w/o_LncMi baseline trains the heterogeneous graph predictor on a reduced network that excludes ncRNA nodes and edges unrelated to the target task. For instance, in the case of lncRNA–drug resistance association prediction task, the network includes only three subnetwroks: lncRNA similarity, drug similarity, and lncRNA–drug resistance association subnetworks. Similarly, for the miRNA–drug resistance association prediction task, only miRNA similarity, drug similarity, and miRNA–drug resistance association subnetworks are retained. The w/o_attribute baseline removes all task-specific attribute features from the nodes and instead uses one-hot encoded vectors as initial node representations, thereby omitting the semantic information introduced through attribute feature encoding. HG_SeqSim replaces the original expression-based ncRNA similarity networks in the heterogeneous graph with sequence-based similarity networks. Specially, lncRNA and miRNA sequences were obtained from RNAcentral (Consortium 2021). These sequences were embedded using Word2Vec (Church 2017), and ncRNA similarity was also computed via Kendall’s tau coefficient.

The comparative results are presented in Table 2, from which we can see the followings: (1) Across both datasets, iNcRD-HG consistently outperforms the three baseline models, achieving the highest AUC and AUPR scores. These results highlight the importance of incorporating semantic information derived from related ncRNA subnetworks and attribute features in enhancing predictive performance; (2) Integrating cross-ncRNA interactions leads to a more pronounced performance improvement in lncRNA–drug resistance prediction than in miRNA–drug resistance prediction. Unlike miRNAs, which act primarily via direct post-transcriptional regulation, lncRNAs often function by competitively binding to miRNAs. Incorporating miRNA–lncRNA regulatory relationships therefore provides additional context that helps iNcRD-HG capture the indirect but important roles of lncRNAs in drug resistance; (3) Using sequence-based ncRNA similarity networks (HG_SeqSim) leads to slightly lower performance than iNcRD-HG, suggesting that expression profiles across cancer cell lines provide additional pathological context beyond sequence information for predicting ncRNA–drug resistance associations.

**Table 2:** Performance comparison of iNcRD-HG with baseline methods on $S_{\mathrm{LD}}^{\mathrm{val}}$ and $S_{\mathrm{MD}}^{\mathrm{val}}$.

Dataset	Method	AUC	AUPR
$S_{\mathrm{LD}}^{\mathrm{val}}$	iNcRD-HG	**0.9834**	**0.9545**
	w/o_LncMi	0.9729	0.9242
	w/o_attribute	0.9699	0.9282
	HG_SeqSim	0.9718	0.9403
$S_{\mathrm{MD}}^{\mathrm{val}}$	iNcRD-HG	**0.9555**	**0.9183**
	w/o_ LncMi	0.9518	0.9098
	w/o_attribute	0.9492	0.9062
	HG_SeqSim	0.9480	0.9084

Bold values indicate the best performance.

### 3.3 Feature analysis of heterogeneous graph representations

**Figure 3:**
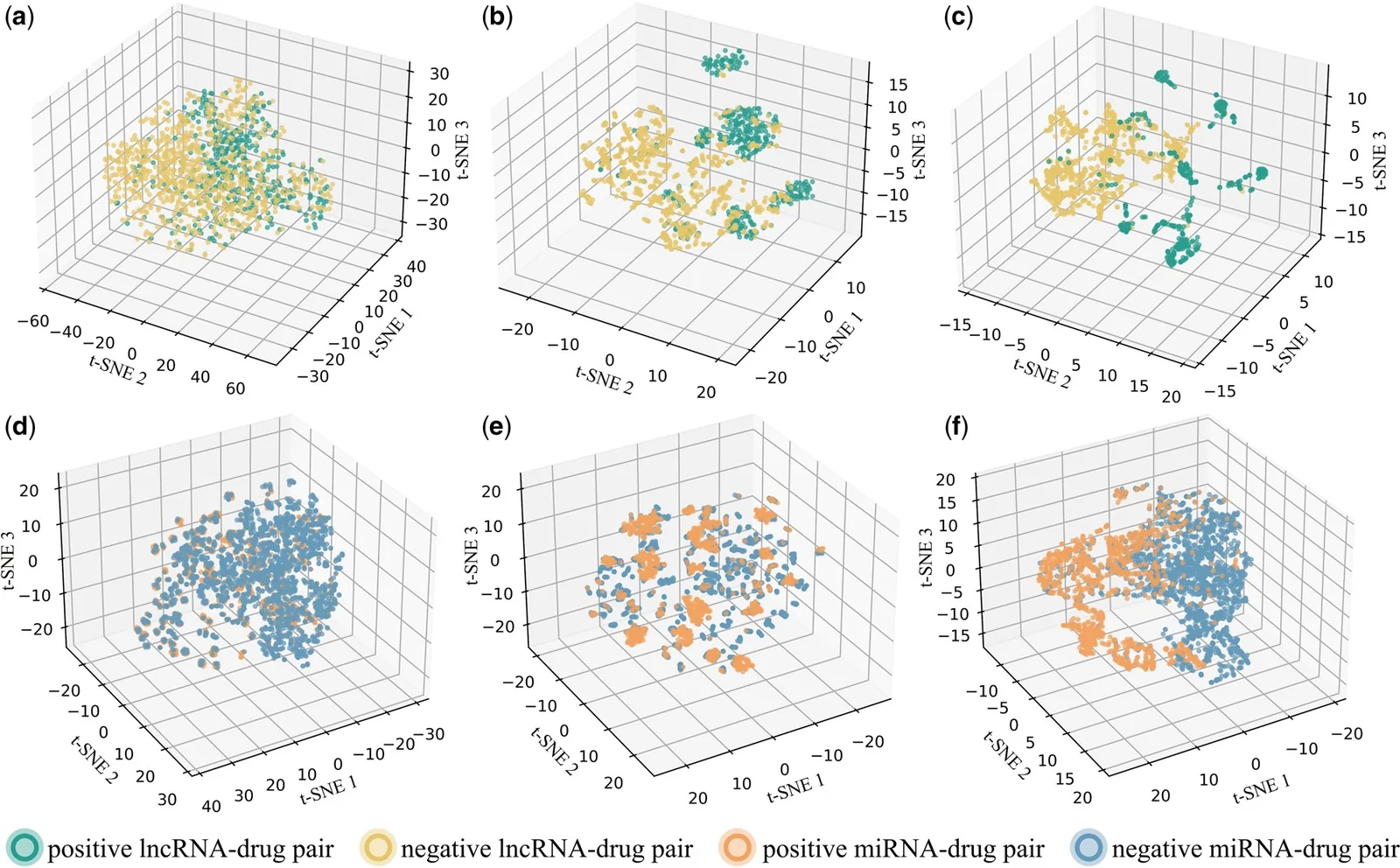
T-SNE visualization of different pair representations. (a, d) Features derived from attribute concatenation. (b, e) Features obtained through structural concatenation. (c, f) Features learned by iNcRD-HG.

To further investigate the discriminative capability of ncRNA–drug resistance pair representations learned by iNcRD-HG, we performed visualization analyses using t-SNE. Three different pair representation strategies were compared. The first strategy, attribute concatenation, involves directly concatenating the attribute features of ncRNA and drug nodes. The second strategy, structural concatenation, integrates similarity vectors derived from ncRNA and drug similarity networks as the pair representation. The third strategy employs the pair representations extracted by iNcRD-HG through its heterogeneous graph learning framework, which jointly encodes node attribute features and topological dependencies across multi-relational molecular networks. As shown in Fig. 3, we visualized the pair representations produced by these three strategies on the testing datasets $S_{\mathrm{LD}}^{\mathrm{test}}$ and $S_{\mathrm{MD}}^{\mathrm{test}}$. The results clearly demonstrate that attribute concatenation and structural concatenation show less separable clusters, whereas the representations learned by iNcRD-HG exhibit superior label separability. This improvement can be attributed to iNcRD-HG ability to integrate intrinsic semantic information from ncRNA and drug attributes with the complex topological semantics of molecular interactions, further enhanced by the supervision provided by association signals during representation learning.

### 3.4 Interpretable predictions of ncRNA–drug resistance associations

**Figure 4:**
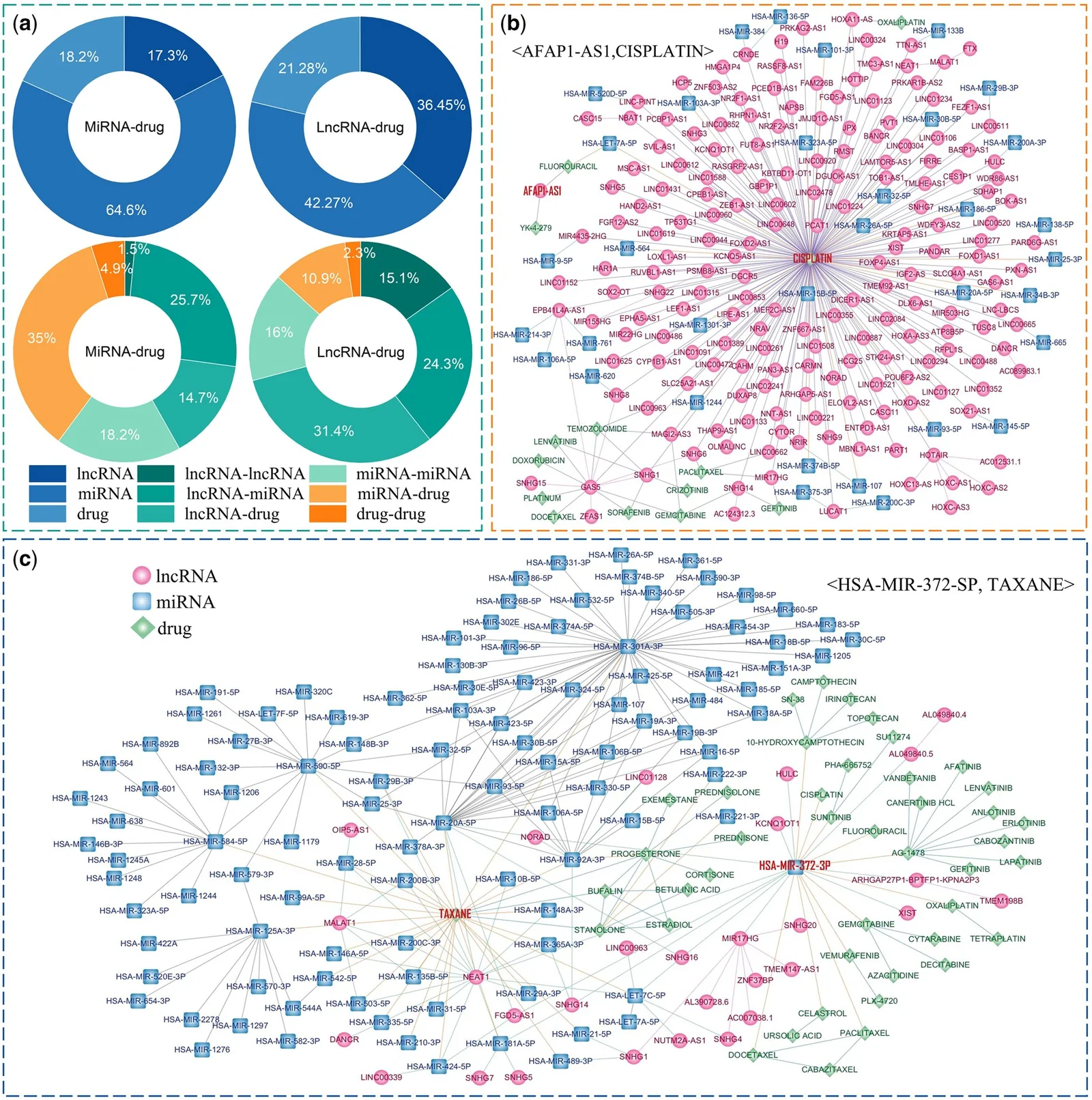
Interpretability analysis of iNcRD-HG predictions. (a) Contribution of different node and edge types in heterogeneous network to iNcRD-HG prediction. (b) Edge-contribution subnetwork for experimentally validated AFAP1-AS1 associated with CISPLATIN resistance. (c) Edge-contribution subnetwork for experimentally validated HSA-MIR-372-3P associated with TAXANE resistance.

To elucidate the inference pathways of iNcRD-HG in predicting ncRNA–drug resistance associations and enhance its biological interpretability for guiding experimental validation, we introduced Captum Explainer (Kokhlikyan *et al.* 2020) to compute attribution scores by back-propagating through iNcRD-HG model. We applied CaptumExplainer to the testing datasets $S_{\mathrm{LD}}^{\mathrm{test}}$ and $S_{\mathrm{MD}}^{\mathrm{test}}$, and evaluated the importance scores of different nodes and edges within the constructed heterogeneous network. Figure 4a summarizes the results and reveals several key findings: (1) Among node types, miRNAs exhibit the highest contribution in both tasks, particularly in miRNA–drug resistance association prediction (64.6%), while lncRNAs contribute more prominently in lncRNA–drug resistance association prediction (36.4%), reflecting the task-specific relevance of different ncRNA types; (2) All edge types contribute to the final prediction, with lncRNA–drug and miRNA–drug edges showing the greatest impact, underscoring the importance of direct ncRNA–drug resistance associations; (3) Notably, lncRNA–miRNA interactions also play a significant role in both tasks (24.3% and 25.7%, respectively), aligning with the known bio logical mechanism of ncRNA cross-talk.

Two experimentally validated associations <AFAP1-AS1, CISPLATIN> and <HSA-MIR-372-3P, TAXANE>, were analysed using CaptumExplainer to identify key molecular components and interactions driving inference, with Fig. 4b and c showing their top-300 edge-contribution subnetworks. For <AFAP1-AS1, CISPLATIN>, iNcRD-HG highlights meta-paths such as <AFAP1-AS1, FLUOROURACIL, HSA-LET-7A-5P, CISPLATIN>, implying that AFAP1-AS1 may exert cisplatin resistance through interactions with shared chemoresistance-associated regulators like let-7a or chemotherapeutic cross-talk pathways. While AFAP1-AS1 has been experimentally shown to promote cisplatin resistance via sponging miR-139-5p and upregulating RRM2 (Huang *et al.* 2019), the inference of let-7a suggests additional or parallel regulatory axes, as let-7a is known to modulate apoptosis-related genes such as BCL2 and HMGA2, potentially converging on similar resistance mechanisms. For <HSA-MIR-372-3P, TAXANE>, several high-impact meta-paths are identified, such as <HSA-MIR-372-3P, SNHG16, HSA-LET-7C-5P, TAXANE> and <HSA-MIR-372-3P, NORAD, HSA-MIR-20A-5P, TAXANE>, suggesting that miR-372-3p may influence taxane resistance through regulatory cascades involving lncRNAs and shared miRNA hubs such as let-7 and miR-20a families. These findings are biologically plausible, as miR-372-3p was found dysregulated in taxane-resistant tumors (Chen *et al.* 2020), SNHG16 has been linked to chemoresistance through modulation of cell cycle regulators, and NORAD contributes to genomic stability by sequestering PUMILIO proteins (Dong *et al.* 2019, Elguindy and Mendell 2021), which may indirectly affect drug response pathways. Two additional validated associations <XIST, OXALIPLATIN> and <HSA-MIR-199B-5P, SORAFENIB> were analysed, and their edge-contribution subnetworks are shown in Supplementary Fig. S2, available as supplementary data at *Bioinformatics* online.

Overall, these results highlight that iNcRD-HG effectively integrates node-level semantic attributes with molecular interaction edges to capture biologically meaningful pathways, while meta-path interpretation elucidates plausible mechanisms of ncRNA–drug resistance and uncovers novel intermediate nodes that may constitute unexplored regulatory axes warranting experimental validation.

### 3.5 Performance comparison with related methods

To evaluate the predictive performance of iNcRD-HG for ncRNA–drug resistance associations, we compared it against nine competing methods. Among these, five are GNN baselines, including GAT (Veličković *et al.* 2018), GraphSAGE (Hamilton *et al.* 2017), ASAP (Ranjan *et al.* 2020), GeneralGNN (You *et al.* 2020), and GraphConv (Morris *et al.* 2019). These GNN-based models utilized the node attribute features extracted in this study and were trained on a biological network comprising ncRNA–drug resistance associations, ncRNA similarity and drug similarity associations, derived by removing non-target ncRNA nodes and their associated edges from our heterogeneous network. The other four competing methods are existing ncRNA–drug association prediction approaches: GCMDR (Huang *et al.* 2020), DeepLDA (Gao and Shang 2023), AGCLNDA (Fan *et al.* 2025), and NDSGCL (Hu *et al.* 2024). GCMDR and DeepLDA leverage ncRNA and drug node attribute features and employ GCN and DNN architectures to extract high-level representations, whereas AGCLNDA and NDSGCL rely on graph contrastive learning to capture topological representations without explicit node attributes. Table 3 summarizes the performance comparison on two testing datasets $S_{\mathrm{LD}}^{\mathrm{test}}$ and $S_{\mathrm{MD}}^{\mathrm{test}}$. The results reveal the key observations: iNcRD-HG consistently outperforms all baselines across both datasets, achieving the highest AUC and AUPR scores, underscoring its ability to integrate attribute information with heterogeneous topological semantics for robust ncRNA–drug resistance association prediction. In contrast, graph contrastive learning methods exhibit unstable performance, particularly in $S_{\mathrm{LD}}^{\mathrm{test}}$, potentially linked to their reliance on topology, making them more vulnerable to noise and reducing generalization. Attribute-based methods and standard GNN models perform competitively but still lag behind.

**Table 3:** Performance comparison of different methods on$\;S_{\mathrm{LD}}^{\mathrm{test}}$ and $S_{\mathrm{MD}}^{\mathrm{test}}$.

Method	$S_{\mathrm{LD}}^{\mathrm{test}}$		$S_{\mathrm{MD}}^{\mathrm{test}}$	
	AUC	AUPR	AUC	AUPR
GAT	0.9652	0.9273	0.8917	0.7219
GraphSAGE	0.9713	0.9202	0.9383	0.8718
ASAP	0.9525	0.8939	0.9160	0.8787
GeneralGNN	0.9615	0.9127	0.9226	0.8644
GraphConv	0.9469	0.9148	0.8989	0.8293
GCMDR	0.9634	0.9430	0.9123	0.8554
DeepLDA	0.9729	0.9335	0.9393	0.8850
AGCLNDA	0.6646	0.7643	0.9244	0.8871
NDSGCL	0.7723	0.7679	0.8008	0.6732
iNcRD-HG	**0.9857**	**0.9585**	**0.9564**	**0.9165**

Bold values indicate the best performance.

### 3.6 Performance under unseen ncRNA or drug conditions

To assess model generalization, we conducted leave-one-ncRNA-out and leave-one-drug-out experiments, and compared iNcRD-HG with six GNN-based baseline methods, including GCN, GAT, GraphSAGE, ASAP, GeneralGNN, and GraphConv, under unseen ncRNA or drug conditions.

**Figure 5:**
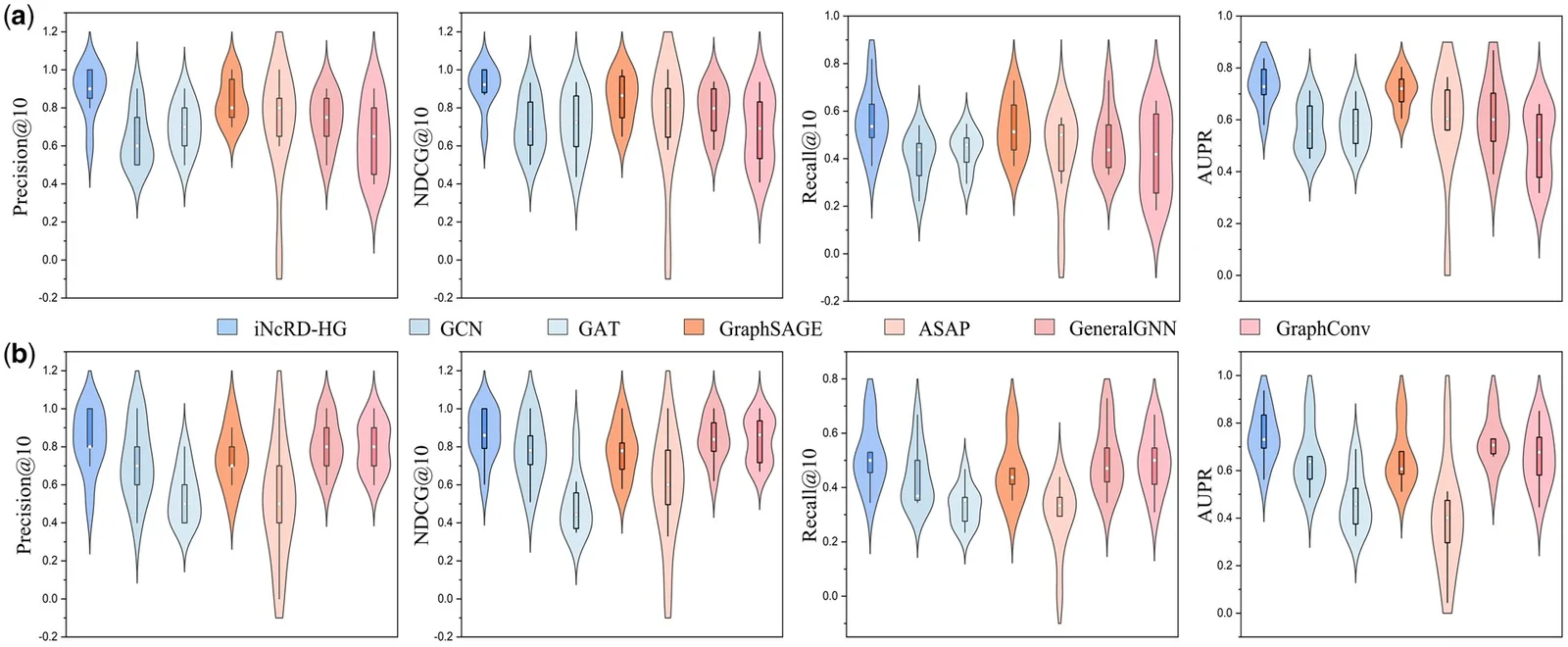
Performance of different methods under unseen ncRNA conditions. (a) Performance distributions for the leave-one-lncRNA-out experiment. (b) Performance distributions for the leave-one-miRNA-out experiment.

For the unseen ncRNA condition, both leave-one-lncRNA-out and leave-one-miRNA-out experiments were performed. LncRNAs and miRNAs with more than 10 known drug resistance associations in the datasets $S_{\mathrm{LD}}^{+}$ and $S_{\mathrm{MD}}^{+}$ were selected for evaluation. The performance distributions across four evaluation metrics obtained by different methods are shown in Fig. 5a and b, respectively. Under the unseen lncRNA setting, iNcRD-HG consistently outperforms all six baseline models across all evaluation metrics. Under the unseen miRNA setting, iNcRD-HG achieves higher AUPR values and exhibits ranking performance comparable to GraphConv, underscoring its ability to prioritize true associations and comprehensively identify potential ncRNA–drug resistance relationships.

**Figure 6:**
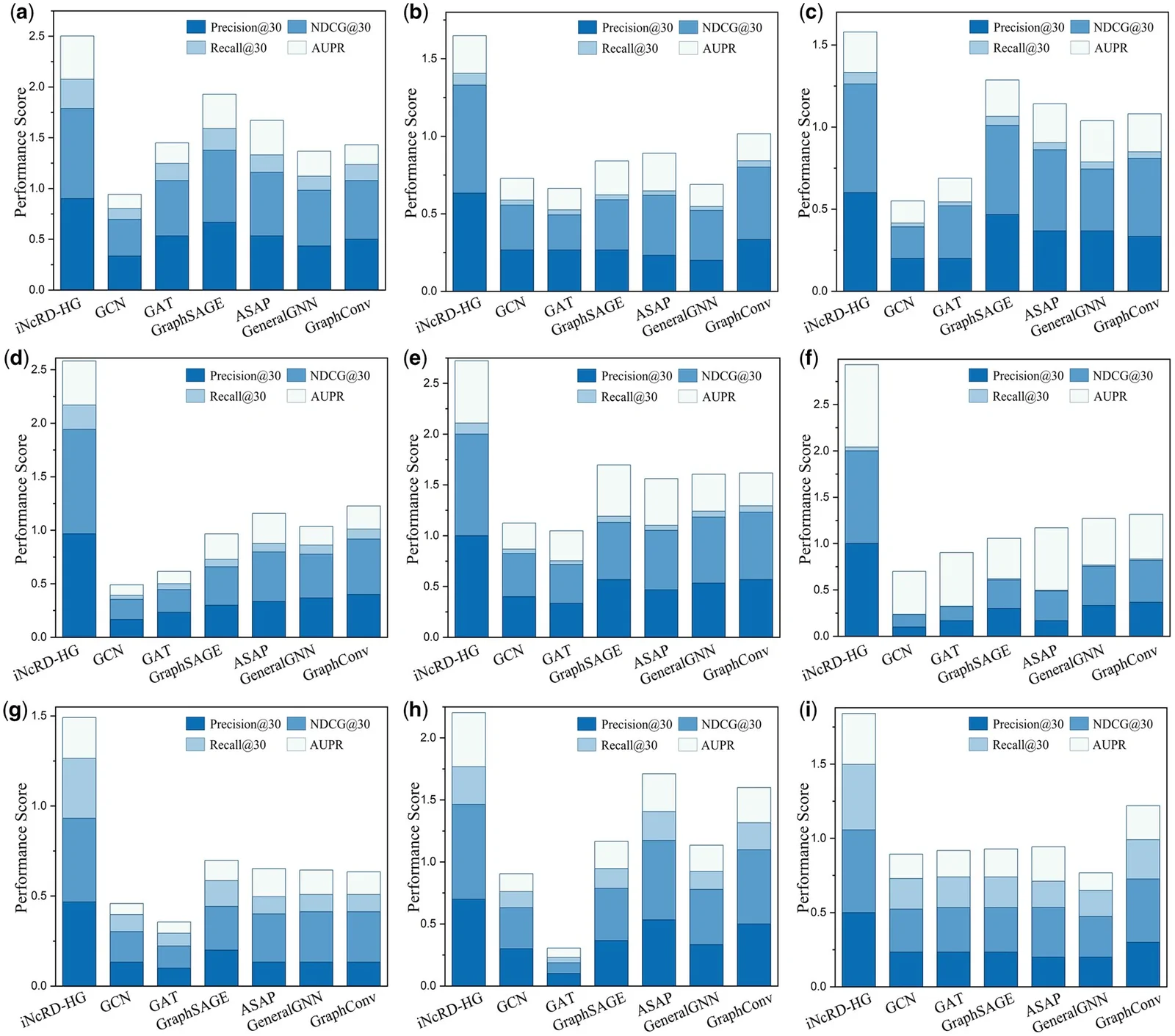
Performance of different methods in leave-one-drug-out experiments on $S_{\mathrm{LD}}$. (a–i) Evaluation metrics under unseen drug scenarios, with Doxorubicin, Oxaliplatin, Fluorouracil, Temozolomide, Cisplatin, Gemcitabine, Gefitinib, Paclitaxel, and Sorafenib each sequentially treated as the simulated novel drug.

**Figure 7:**
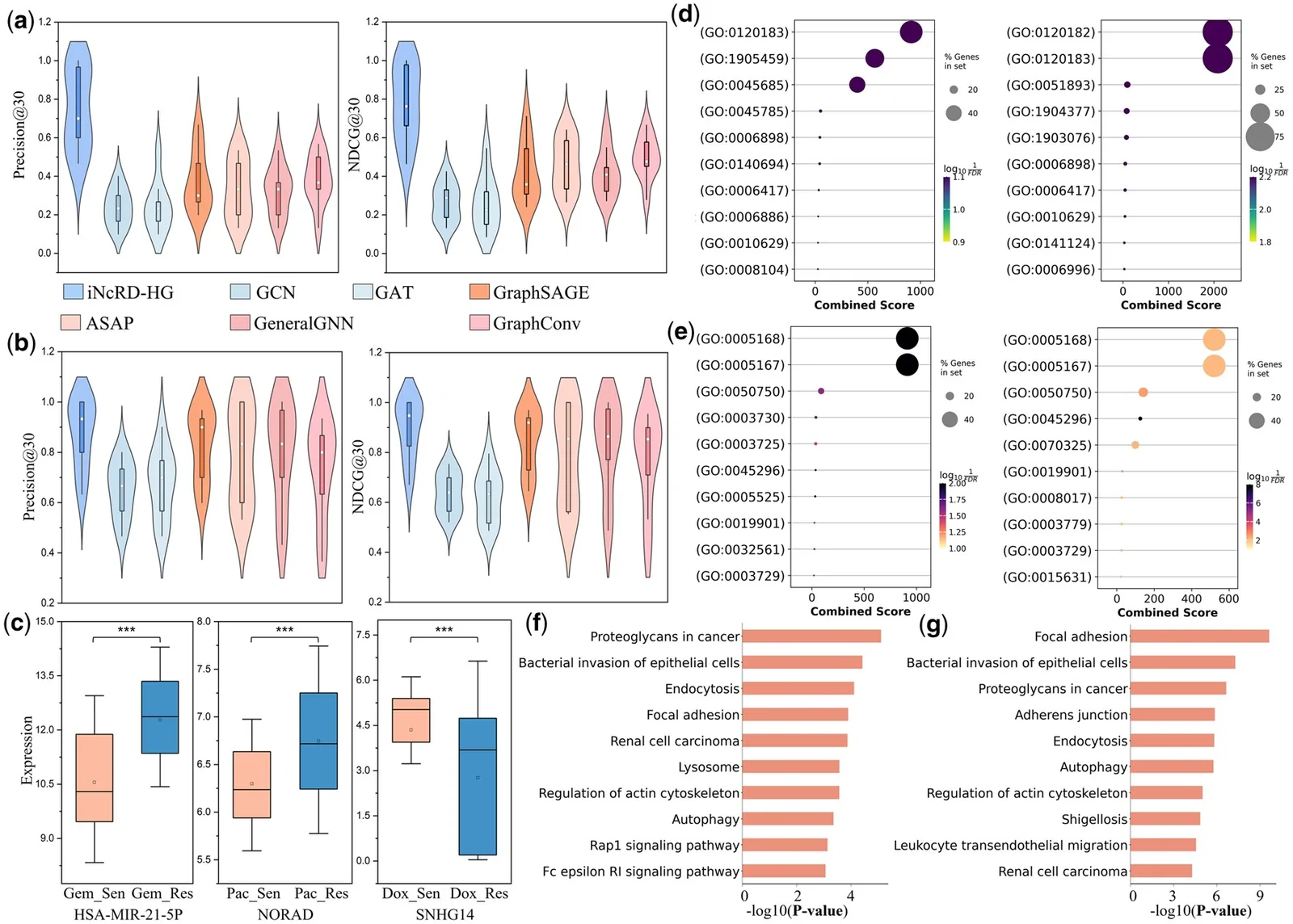
Case study analysis of iNcRD-HG under simulated novel drug scenarios. (a and b) Leave-one-drug-out performance metrics for lncRNA- (a) and miRNA- (b) drug resistance association prediction tasks across different methods. (c) Expression levels of three predicted ncRNAs in drug-sensitive and drug-resistant cells for Gemcitabine (Gem), Paclitaxel (Pac), and Doxorubicin (Dox), where ‘_Sen’ and ‘_Res’ indicate drug-sensitive and drug-resistant cells, respectively. (d) GO Biological Process (BP) enrichments of ncRNAs associated with Gemcitabine and Paclitaxel resistance identified by iNcRD-HG. (e) GO Molecular Function (MF) enrichments of ncRNAs associated with Gemcitabine and Paclitaxel resistance identified by iNcRD-HG. (f and g) KEGG pathway enrichments of ncRNAs associated with Gemcitabine and Paclitaxel resistance identified by iNcRD-HG.

For leave-one-drug-out experiment, nine chemotherapeutic drugs were selected for evaluation, as each exhibits more than 30 known ncRNA resistance associations in the datasets $S_{\mathrm{LD}}^{+}$ and $S_{\mathrm{MD}}^{+}$. The drug-specific results are analyzed and illustrated in Fig. 6, from which we can see that iNcRD-HG achieves the best performance across all four metrics compared to six GNN-based baseline methods, with particularly notable improvements in Precision@30 and NDCG@30. This demonstrates iNcRD-HG can accurately prioritize true ncRNA-drug resistance associations and achieve higher ranking quality. From an overall perspective, all methods show generally higher Precision@30 and NDCG@30 compared to Recall@30. This phenomenon can be attributed to the limited number of known ncRNA-drugs associations: with relatively few true positives per drug and a fixed k value of 30, ranking precision and quality are more readily reflected, whereas recall are inherently constrained by the small pool of true associations. Comparative results of miRNA-drug prediction performance across the nine unseen drugs are provided in Supplementary Fig. S3, available as supplementary data at *Bioinformatics* online. The performance distributions of lncRNA-drug and miRNA-drug resistance association prediction are shown in Fig. 7a and b, with detailed mean metrics provided in Supplementary Tables S2 and S3, available as supplementary data at *Bioinformatics* online. iNcRD-HG outperforms all baselines in both tasks, achieving superior Precision@30 and NDCG@30, underscoring its ability to rank true associations at the top positions. These results demonstrate that iNcRD-HG excels not only in standard prediction scenarios but also maintains robustness under different cold-start conditions.

### 3.7 Case study

To further illustrate the predictive capability of iNcRD-HG under unseen drug conditions, we conducted case studies on three representative drugs including Gemcitabine, Paclitaxel, and Doxorubicin. Table 4 lists the top 10 lncRNAs and miRNAs predicted by iNcRD-HG as resistance-associated candidates for each drug. Of the top-ranked candidates, 26 lncRNAs and 30 miRNAs are supported by experimental evidence reported in the literature. For example, knockdown of NEAT1 reverses paclitaxel resistance in non-small cell lung cancer (NSCLC) cell lines (Li *et al.* 2019), inhibition of miR-21 increases sensitivity to gemcitabine (Meng *et al.* 2006), and NORAD promotes proliferation and metastasis while conferring doxorubicin resistance and inhibiting apoptosis and autophagy in neuroblastoma cells (Wang *et al.* 2020). Certain ncRNAs, including MALAT1, XIST, and HSA-MIR-34A-5P, appear recurrently across different drugs, suggesting their potential roles as shared regulators of multidrug resistance. Although several predicted ncRNAs lack direct experimental validation, NORAD and SNHG14 showed significant differential expression in paclitaxel- and doxorubicin-resistant versus sensitive cells, respectively (Fig. 7c). Together, these findings highlight that iNcRD-HG can not only recover known drug resistance-associated ncRNAs but also identify promising candidates with potential clinical relevance.

**Table 4:** The top ten ncRNAs associated with different drug resistance predicted by iNcRD-HG.

Drug	lncRNA	Evidence	miRNA	Evidence
Gemcitabine	AP001043.1	33436545	HSA-MIR-21-5P	16762633
	HAO2-IT1	33436545	HSA-MIR-34A-5P	21347785
	LINC01093	33436545	HSA-MIR-200C-3P	19654291
	LINC01831	33436545	HSA-MIR-181A-5P	28560603
	CPS1-IT1	33436545	HSA-MIR-126-3P	31514732
	AL031722.1	33436545	HSA-MIR-375-3P	31514732
	AC022784.3	33436545	HSA-MIR-203A-3P	24040438
	LINC01595	33436545	HSA-MIR-210-3P	25117811
	AC008549.1	33436545	HSA-MIR-145-5P	27765914
	AL355997.1	33436545	HSA-MIR-100-5P	23946872
Paclitaxel	NEAT1	30782035	HSA-MIR-93-5P	30244336
	XIST	12492109	HSA-MIR-16-5P	20460378
	MALAT1	31837057	HSA-MIR-15A-5P	23826416
	KCNQ1OT1	28600629	HSA-MIR-26B-5P	29125598
	H19	29968942	HSA-MIR-103A-3P	30774764
	OIP5-AS1	29219179	HSA-MIR-101-3P	24755562
	SNHG16	31287002	HSA-MIR-32-5P	24220856
	HOTAIR	29680837	HSA-MIR-301A-3P	26323677
	UCA1	29777711	HSA-MIR-25-3P	26323677
	NORAD	unconfirmed	HSA-MIR-106B-5P	20460378
Doxorubicin	NEAT1	30349370	HSA-MIR-21-5P	19412672
	XIST	30907503	HSA-MIR-34A-5P	18645025
	MALAT1	27524242	HSA-MIR-200C-3P	19412672
	KCNQ1OT1	31837329	HSA-MIR-181A-5P	19412672
	SNHG16	unconfirmed	HSA-MIR-126-3P	19237188
	NORAD	32563146	HSA-MIR-146A-5P	19412672
	SNHG14	unconfirmed	HSA-MIR-155-5P	18971180
	OIP5-AS1	30204936	HSA-MIR-29A-3P	18645025
	FGD5-AS1	unconfirmed	HSA-MIR-29B-3P	18645025
	H19	8674037	HSA-MIR-451A	19412672

Potential biological mechanisms by which ncRNAs contribute to drug resistance were investigated through functional enrichment analysis of the top 100 candidates predicted by iNcRD-HG. As shown in Fig. 7d, ncRNAs associated with gemcitabine resistance are significantly enriched in GO Biological Process (BP) terms such as focal adhesion disassembly, apoptosis regulation, receptor-mediated endocytosis, and translational control, indicating that alterations in adhesion dynamics, intracellular transport, and gene expression regulation are central to gemcitabine response. In contrast, paclitaxel-related ncRNAs are enriched in processes involving focal adhesion assembly/disassembly, protein localization, intracellular signalling, and biosynthetic regulation, highlighting a stronger involvement of signalling modulation and membrane protein trafficking in paclitaxel resistance. Molecular function analysis (Fig. 7e) indicates common enrichment in cadherin binding, protein kinase binding, and mRNA binding, but gemcitabine resistance uniquely involved GTP and double-stranded RNA binding, while paclitaxel resistance is characterized by microtubule, tubulin, and actin binding, reflecting its mechanism as a microtubule-stabilizing drug. KEGG pathway analysis (Fig. 7f–g) indicates overlapping involvement of focal adhesion, endocytosis, autophagy, and actin cytoskeleton regulation, alongside drug-specific pathways such as Rap1 and Fc epsilon RI signalling for gemcitabine, and adherens junction and leukocyte transendothelial migration for paclitaxel. These results suggest that ncRNAs contribute to chemoresistance via shared pathways regulating adhesion, endocytosis, and cytoskeletal remodelling, while also engaging distinct biological processes aligned with each drug pharmacological action.

## 4. Conclusion

Deciphering how non-coding RNAs contribute to drug resistance is pivotal for revealing cellular response mechanisms and advancing targeted cancer therapies. In this work, we propose iNcRD-HG, a heterogeneous graph learning framework that integrates multi-source biological information, including molecular interactions, ncRNA expression patterns, and drug structural features, to construct a biologically enriched network. By leveraging relation-type-aware message passing and an interpretability module, iNcRD-HG captures intricate contextual relationships and uncovers cooperative ncRNA effects influencing drug resistance. Experimental results demonstrate iNcRD-HG achieves superior predictive performance compared with existing methods, effectively uncovering synergistic ncRNA interactions and biologically meaningful pathways associated with drug resistance.

Although iNcRD-HG demonstrates promising results, several avenues for improvement remain: (i) Incorporating multiple ncRNA modalities, including sequence and expression features, along with the validated circRNA–drug resistance associations, can enhance the representation of ncRNA and enable a more comprehensive exploration of ncRNA-mediated regulatory mechanisms. (ii) A sophisticated multi-task learning module can be introduced to extend iNcRD-HG, as naive multi-task designs may cause task interference. Potential strategies include cross-task consistency regularization leveraging known cross-ncRNA interactions, task-aware contrastive objectives, and adaptive loss reweighting to better preserve task-specific biological signals while regulating cross-task influence. (iii) Since iNcRD-HG is built on aggregated public databases, it currently provides coarse-grained drug resistance associations. Tumor-type-specific or single-cell information can be further integrated to improve tumor–specific interpretation.

## Supplementary Material

btag029_Supplementary_Data

## Data Availability

Datasets and source codes are available at https://github.com/Biohang/iNcRD-HG.
