# Correction to: Semantic-enhanced heterogeneous graph learning for identifying ncRNAs associated with drug resistance

**DOI:** 10.1093/bioinformatics/btag497

**Published:** 2026-08-24

This is a correction to: Hang Wei, Yuran Xie, Wenxiang Zhang, Linyang Li, Shuai Wu, Lin Gao, Semantic-enhanced heterogeneous graph learning for identifying ncRNAs associated with drug resistance, *Bioinformatics*, Volume 42, Issue 2, February 2026, btag029, https://doi.org/10.1093/bioinformatics/btag029

In the originally published version of this manuscript, the corresponding authorship designation was omitted for Shuai Wu and Lin Gao.

This has been corrected so that Hang Wei, Shuai Wu, and Lin Gao are listed as co-corresponding authors.

