# Supplementary material for: Semantic-enhanced heterogeneous graph learning for identifying ncRNAs associated with drug resistance
Source: Bioinformatics. 2026 Jan 14;42(2):btag029. doi: 10.1093/bioinformatics/btag029 (PMC12866671; doi:10.1093/bioinformatics/btag029)
Supplement: btag029_Supplementary_Data [file btag029_supplementary_data.pdf]

Supplementary Information

1. Supplementary Method

To comprehensively assess the performance of different predictors, we employed two global metrics and three top-k ranking metrics. The global metrics include the Area Under the Receiver Operating Characteristic Curve (AUC) and the Area Under the Precision–Recall Curve (AUPR). AUC evaluates the trade-off between the true positive rate (TPR) and false positive rate (FPR) across all classification thresholds:

$$\mathrm{TPR} = \frac{\mathrm{TP}}{\mathrm{TP} + \mathrm{FN}}$$

$$\mathrm{FPR} = \frac{\mathrm{FP}}{\mathrm{FP} + \mathrm{TN}}$$

AUPR emphasizes the balance between Precision and Recall, particularly in imbalanced datasets:

$$\mathrm{Precision} = \frac{\mathrm{TP}}{\mathrm{TP} + \mathrm{FP}}$$

$$\mathrm{Recall} = \frac{\mathrm{TP}}{\mathrm{TP} + \mathrm{FN}}$$

To further evaluate practical relevance, we calculated three top-k ranking metrics for the highest-confidence predictions: Precision@k, normalized discounted cumulative gain (NDCG@k), and Recall@k. These are defined as:

$$\mathrm{Precision@}k = \frac{\#\text{ of true positives in top} - k}{k}$$

$$\mathrm{Recall@}k = \frac{\#\text{ of true positives in top} - k}{\text{Total positives}}$$

$$\mathrm{NDCG@}k = \frac{\mathrm{DCG@}k}{\mathrm{IDCG@}k}$$

$$\mathrm{DCG@}k = \sum_{i=1}^{k} \frac{2^{rel_i} - 1}{\log_2(i + 1)}$$

where TP, FP, TN, FN represent true positives, false positives, true negatives, and false negatives, respectively; $rel_i = 1$ if the i-th prediction is correct, otherwise 0; IDCG@k is the ideal DCG@k. These metrics collectively provide a thorough evaluation of predictive performance under both global and top-ranked scenarios.

2. Supplementary Tables

Table S1. Statistic information of each sub-dataset

Label	Split	#Pairs ($\mathbb{S}_{LD}$)	#Pairs ($\mathbb{S}_{MD}$)
Positive pair	Train	$\mathbb{S}_{LD}^{train+}$: 1267	$\mathbb{S}_{MD}^{train+}$: 3238
	Validate	$\mathbb{S}_{LD}^{val+}$: 421	$\mathbb{S}_{MD}^{val+}$: 1078
	Test	$\mathbb{S}_{LD}^{test+}$: 421	$\mathbb{S}_{MD}^{test+}$: 1078
Negative pair	Train	$\mathbb{S}_{LD}^{train-}$: 2534	$\mathbb{S}_{MD}^{train-}$: 6476
	Validate	$\mathbb{S}_{LD}^{val-}$: 842	$\mathbb{S}_{MD}^{val-}$: 2156
	Test	$\mathbb{S}_{LD}^{test-}$: 842	$\mathbb{S}_{MD}^{test-}$: 2156

Table S2. Performance of different methods in leave-one-drug-out experiments on $\mathbb{S}_{LD}$

Method	Precision@30	NDCG@30	Recall@30	AUPR
GCN	0.2370	0.2641	0.0755	0.1776
GAT	0.2407	0.2606	0.0715	0.1989
GraphSAGE	0.3741	0.4190	0.1057	0.2753
ASAP	0.3296	0.4579	0.0978	<u>0.3254</u>
GeneralGNN	0.3222	0.4141	0.0865	0.2382
GraphConv	<u>0.3815</u>	<u>0.4955</u>	<u>0.1095</u>	0.2513
iNcRD-HG	**0.7519**	**0.7787**	**0.2096**	**0.4264**

Table S3. Performance of different methods in leave-one-drug-out experiments on $\mathbb{S}_{MD}$

Method	Precision@30	NDCG@30	Recall@30	AUPR
GCN	0.6519	0.6370	0.0841	0.5083
GAT	0.6741	0.6203	0.0857	0.5641
GraphSAGE	<u>0.8296</u>	<u>0.8478</u>	<u>0.1076</u>	**0.6539**
ASAP	0.7852	0.7924	0.1004	0.5513
GeneralGNN	0.7963	0.8257	0.0988	0.6164
GraphConv	0.7296	0.7724	0.0896	0.5874
iNcRD-HG	**0.8741**	**0.8937**	**0.1135**	<u>0.6384</u>

3. Supplementary Figures

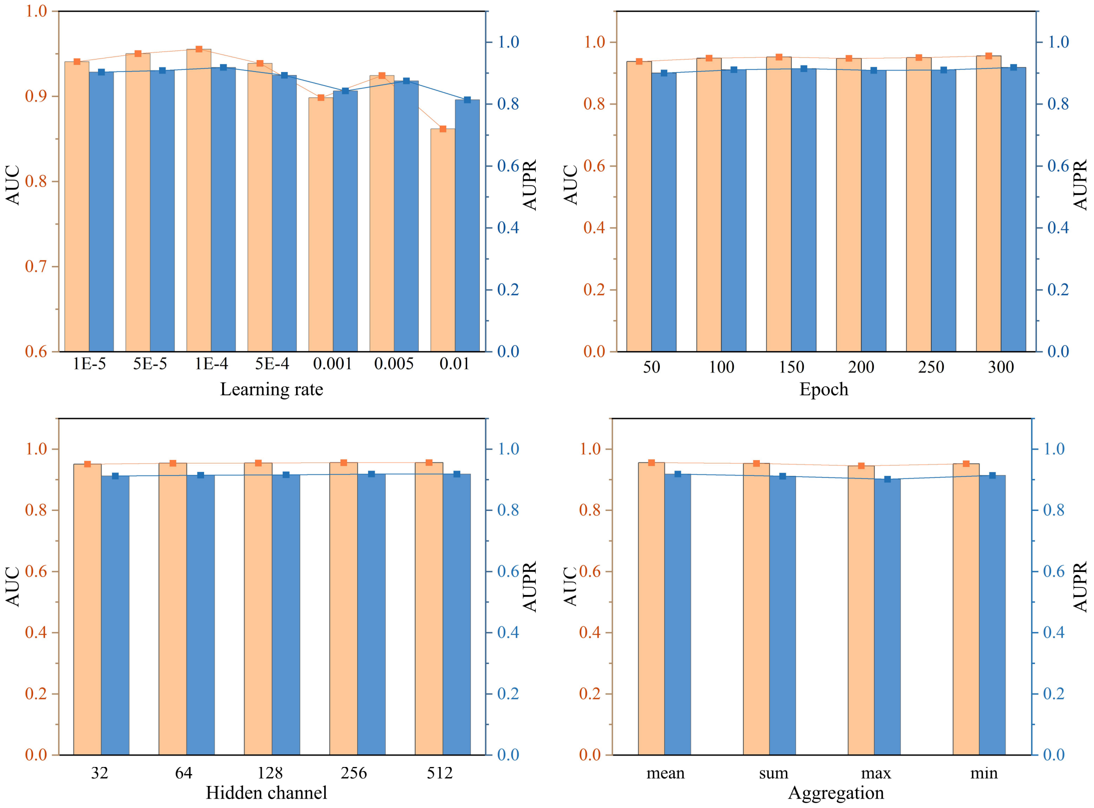

Fig. S1. Parameter analysis of iNcRD-HG. AUC and AUPR obtained with different learning rates, training epochs, hidden dimensions and aggregation strategies on $\mathbb{S}_{\mathrm{MD}}^{\mathrm{val}}$.

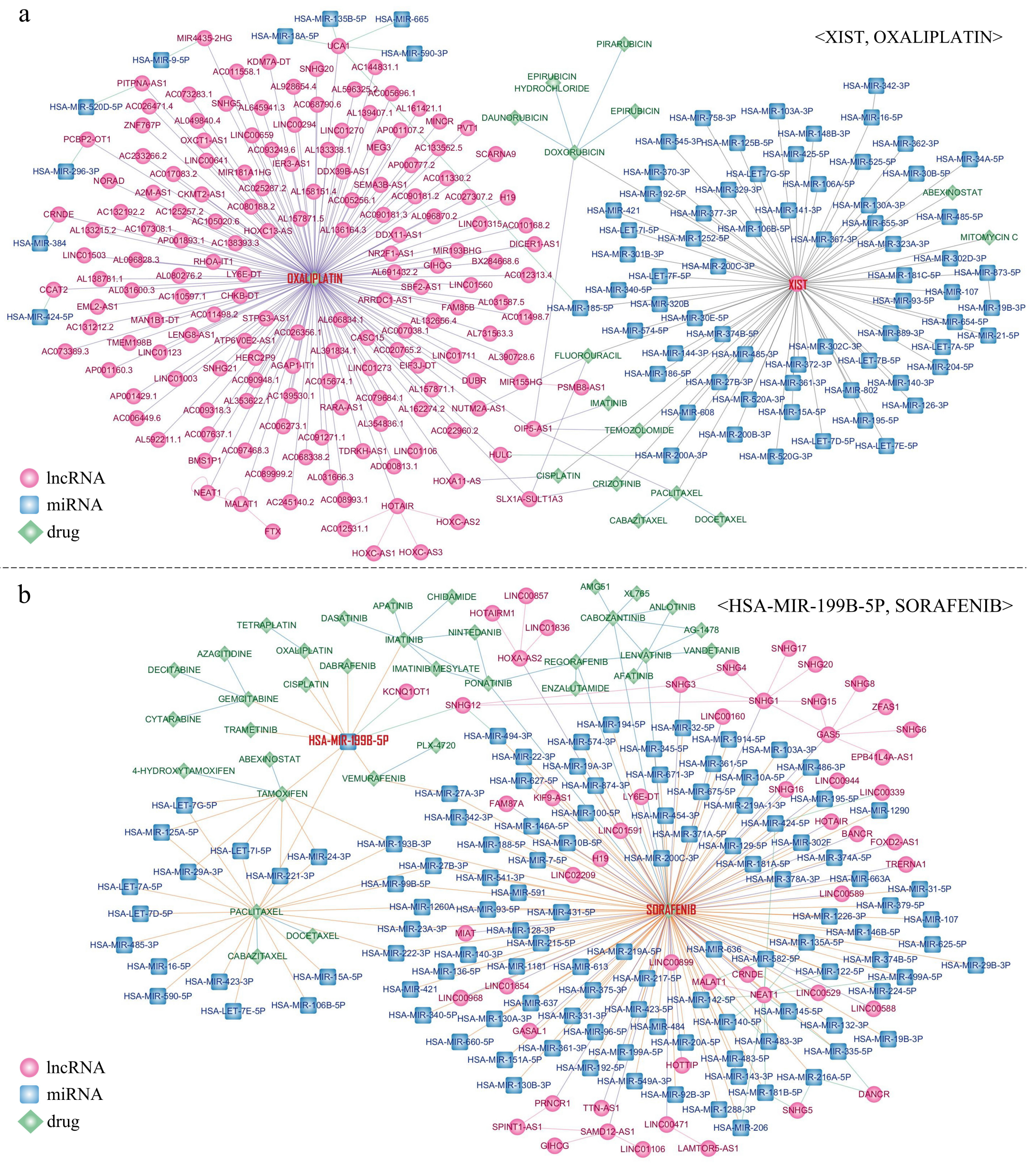

Fig. S2. Edge-contribution subnetworks for experimentally validated ncRNA–drug resistance cases identified by iNcRD-HG. a Edge-contribution subnetwork for experimentally validated XIST associated with OXALIPLATIN resistance. **b** Edge-contribution subnetwork for experimentally validated HAS-MIR-199B-5P associated with SORAFENIB resistance.

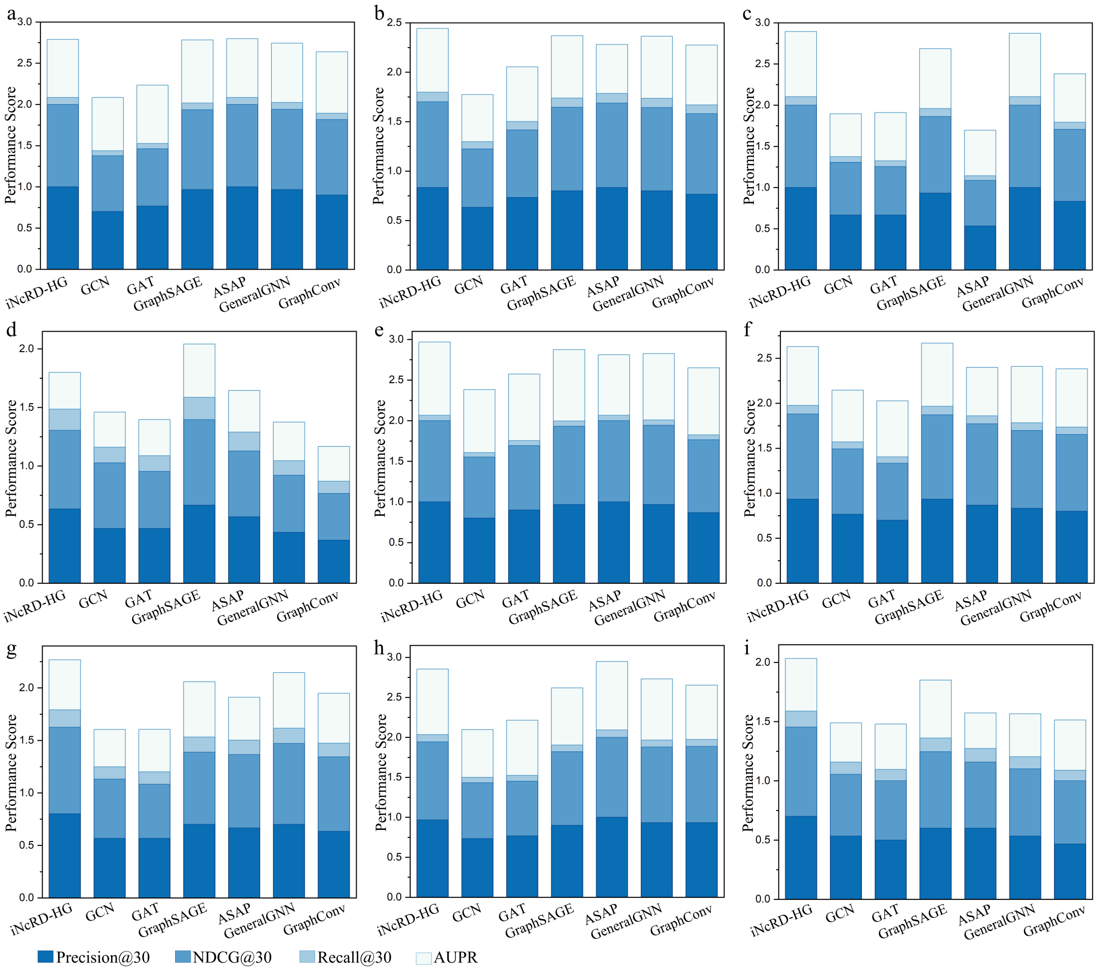

Fig. S3. Performance of different methods in leave-one-drug-out experiments on $\mathbb{S}_{\mathrm{MD}}$. a-i Evaluation metrics under unseen drug scenarios, with Doxorubicin, Oxaliplatin, Fluorouracil, Temozolomide, Cisplatin, Gemcitabine, Gefitinib, Paclitaxel, and Sorafenib each sequentially treated as the simulated novel drug.
